# Development of efficient electroactive biofilm in urine-fed microbial fuel cell cascades for bioelectricity generation

**DOI:** 10.1016/j.jenvman.2019.109992

**Published:** 2020-03-15

**Authors:** Oluwatosin Obata, Maria J. Salar-Garcia, John Greenman, Halil Kurt, Kartik Chandran, Ioannis Ieropoulos

**Affiliations:** a Bristol BioEnergy Centre, Bristol Robotics Laboratory, University of the West of England, BS16 1QY, UK; b Biological, Biomedical and Analytical Sciences, University of the West of England, BS16 1QY, UK; c Department of Earth and Environmental Engineering, Columbia University, NY, USA

**Keywords:** Urine, Microbial fuel cell, Electroactive bacteria, Microbial community structure

## Abstract

The Microbial fuel cell (MFC) technology harnesses the potential of some naturally occurring bacteria for electricity generation. Digested sludge is commonly used as the inoculum to initiate the process. There are, however, health hazards and practical issues associated with the use of digested sludge depending on its origin as well as the location for system deployment. This work reports the development of an efficient electroactive bacterial community within ceramic-based MFCs fed with human urine in the absence of sludge inoculum. The results show the development of a uniform bacterial community with power output levels equal to or higher than those generated from MFCs inoculated with sludge. In this case, the power generation begins within 2 days of the experimental set-up, compared to about 5 days in some sludge-inoculated MFCs, thus significantly reducing the start-up time. The metagenomics analysis of the successfully formed electroactive biofilm (EAB) shows significant shifts between the microbial ecology of the feeding material (fresh urine) and the developed anodic biofilm. A total of 21 bacteria genera were detected in the urine feedstock whilst up to 35 different genera were recorded in the developed biofilm. Members of *Pseudomonas* (18%) and *Anaerolineaceae* (17%) dominate the bacterial community of the fresh urine feed while members of *Burkholderiaceae* (up to 50%) and *Tissierella* (up to 29%) dominate the anodic EAB. These results highlight a significant shift in the bacterial community of the feedstock towards a selection and adaptation required for the various electrochemical reactions essential for survival through power generation.

## Highlights

• Possibility of starting an MFC systems with urine as both inoculum and feedstock.
• Stepwise adjustment of external resistance resulted in the formation of efficient EAB.
• Bacteria community detected in developed anode possess diverse functionalities.
• Inherent factors resulted in the selection, adaptation and enrichment of strains.
• Variations in bacterial community of individual MFCs within the cascade.

## 1 Introduction

Microbial fuel cells (MFCs) are an innovative and environmentally friendly technology that can directly convert waste rich in organics into electricity with a dual benefit: i) bioenergy production and ii) waste treatment. In MFCs, bacteria act as biocatalysts and carry out the conversion of the chemical energy stored in a specific substrate into electrical energy. Cations such as protons and electrons are released in the anodic chamber during the organic matter oxidation process. Protons cross a semipermeable separator to reach the open-air cathode, where they combine with the electrons, coming from the anode, to form water. Redox reactions are completed by the reduction of an oxidant, usually oxygen due to its natural availability and high reduction potential (Allen and Bennetto, 1993; Bruce E. Logan et al., 2006; Potter, 1911). However, a catalyst is still needed to accelerate the oxygen reduction reaction at the cathode. Previous reports have shown that inorganic materials are the most suitable for catalysing the cathodic reaction and can be grouped into: i) platinum group metals (PGMs), ii) metal-free carbonaceous-based materials and iii) platinum group metal-free materials. In laboratory scale systems, the most commonly used catalysts are platinum group metals due to their high performance but their high cost and need for replacement after a time make their use in scaled-up MFCs infeasible (Santoro et al., 2017; Wang et al., 2017).

MFCs have gained much attention over the past decades due to the need to produce cleaner energy as well as develop more sustainable waste management systems (Santoro et al., 2017). Despite much of the interest in the technology being motivated by the depletion of fossil fuels and the quest for better wastewater management, there are currently many other applications where MFCs could be implemented (Ieropoulos et al., 2017) due to more stringent water discharge limits (He et al., 2017). So far, most research work focusses on the use of simple substrates, such as glucose or acetate in MFCs for bioenergy production. However, the main advantage of these bioelectrochemical systems, over other technologies is their ability to directly convert wastewater of different compositions into electricity without any need for power input (Santoro et al., 2017). Brewery wastewater, food processing wastewater, dairy wastewater or swine wastewater are some of the substrates used in MFCs thus far (Pandey et al., 2016; Pant et al., 2010). More recently, the use of neat human urine as the sole feedstock has been reported (Ieropoulos, 2011) with applications such as charging smartphones (Ieropoulos et al., 2017, 2013; Merino et al., 2016; Walter et al., 2016b) and powering lights in toilets (Ieropoulos et al., 2016; Walter et al., 2018). This technology also brings benefits for addressing energy and environmental issues particularly in developing and remote areas of the world, where the household waste is the most available substrate (Ajayi and Weigele, 2012; Rabaey and Verstraete, 2005).

Despite the significant potential of MFCs, their large-scale commercialisation is still hindered by the cost of the electrode material and semipermeable separator, the set-up design or the operating conditions, among others. To address these challenges, substantial efforts have been dedicated in the last few years to new materials and configuration designs in order to optimise the power output and reduce the overall cost of the devices. Low cost materials, such as carbon-based electrodes or ceramic-based membranes, have been successfully reported as feasible and cost-effective alternatives for MFCs. Their natural availability, high biocompatibility and low price, bring meaningful advantages to the real implementation of this technology (Chen et al., 2019; Mateo et al., 2018; Roustazadeh Sheikhyousefi et al., 2017).

As MFC applications move from the laboratory to the field, the practicalities of implementation continue to pose a challenge particularly at the start-up phase and especially when deployment will take place in remote rural places, where logistical challenges would likely arise. One such challenge is the need for initial sludge inoculation (Merino et al., 2016). Availability and suitability of sludge, which is safe and screened for pathogens for the inoculation stage poses a challenge, as it is currently difficult to find in the desired quantities. This problem could hinder the widespread implementation of this technology in areas that need it the most. In this context, the present work investigates the effect of the inoculation stage on both the diversity of the anodic microbial community and the power output by ceramic MFCs working in cascade. One of the inoculation methods proposed involves a sludge-free inoculation process to evaluate the impact onto the performance of ceramic MFCs fed with human urine. Most of the work reported in the literature thus far mainly focusses on the physical characterisation of the materials, but information about the bacterial communities is limited. Taking into account that bacteria are the main drivers of this technology, it is essential to understand the impact of the inoculation procedure on the anodic bacterial diversity and therefore on the power output by the MFC.

## 2 Materials and methods

### 2.1 MFC reactor construction and operation

MFCs were assembled using terracotta cylinders sealed at one end (Orwell Aquatics, UK) with the following dimensions: length 5 cm, outside diameter 2.9 cm, inside diameter 2.1 cm, wall thickness 4 mm. The anode electrode was made of carbon veil (carbon loading 20 g m^−2^) with a macro surface area of 300 cm^2^, which was folded and wrapped around the terracotta cylinder using nickel chromium (Ni–Cr) wire for current collection. The cathode was made of activated carbon (30% wet proofed with PTFE) as previously described (Gajda et al., 2015). The 30 cm^2^ activated carbon-coated cathode was inserted into the cylinder and connected using a stainless-steel crocodile clip. The whole reactor was placed in a plastic container (60 mL working volume) where the outer anode surface was fully immersed into the anolyte. Ni–Cr wire was used to connect both electrodes to the multi-channel Agilent 34972A (Farnell, UK) logging device and the electrical load. The set-up included nine MFCs configured in cascade and fed with only fresh human urine from the start of the experiment at a flow rate of 20 mL h^−1^ and under a variable external loading regime (from this point onwards, labelled as A1-A9). The experimental set-up was designed as previously described (Ieropoulos et al., 2019).

### 2.2 Inoculation methods

To evaluate the feasibility of starting urine-fed MFCs system without the use of sludge, a stepwise external resistance application approach was adopted. The MFC anodes had no prior inoculation and were started using neat human urine with pH ranging between 6.5 and 7.1, at a flow rate of 20 mL h^−1^, followed by sequential adjustment of the external resistance. At the start of the operation, all MFCs were operated in open circuit voltage (OCV) for 24 h and then, an external resistance of 700 Ω was applied. This was the start of power generation, which resulted in a gradual increase in power. The external loading was changed from 700 Ω to 200 Ω every 7 days to allow for optimal acclimatisation of the anodic bacterial community The final external load applied to the MFC cascade was 100 Ω, which was a value determined from polarisation experiments.

### 2.3 Performance measurements

The voltage of the two set-ups was continuously monitored by a multi-channel Agilent 34972A (Farnell, UK) data logging device. An automatic resistorstat tool was used to perform the polarisation test by varying the external loading from 999,999 to 0 Ω (including open circuit voltage). The urine before and after being treated in the MFCs was characterised by measuring its pH and conductivity with a Hanna 8424 pH meter (Hanna, UK) and a Jenway conductivity meter (Camlab, UK) with an operating range of 0–1999 mS cm^−1^, respectively.

### 2.4 DNA isolation, next-generation 16S rRNA amplicon sequencing and sequence data analysis

After 80 days of operation, anodic biofilm bacteria were collected and analysed. Metagenomic DNA from samples were extracted in duplicates using Dneasy Blood & Tissue kit (Qiagen, Germantown, MD) according to the kit protocol. DNA quality and quantity were measured by Nano-Drop Lite spectrophotometer (Thermo Fisher Scientific, Waltham, MA, U.S.A.). Bacterial 16S rRNA gene was amplified using universal primers 1055f (ATGGCTGTCGTCAGCT) and 1392r (ACGGGCGGTGTGTAC) (Ferris and Muyzer, 1996) and barcoded fusion primers with sequencing adaptors (Park et al., 2017a). The quality and quantity of the 16S amplicon sequence was checked with a bioanalyser (Agilent Technologies 2100, CA, U.S.A.). 16S amplicon sequencing was performed using an Ion Torrent PGM (Thermo Fisher, MA, U.S.A.) platform with Ion Torrent 318v2 Ion Chip by following the manufacturer's instructions (Ion PGM Hi-Q Sequencing kit, Product no. MAN0009816). All 16S rRNA gene raw sequences have been deposited to Sequence Read Archive (SRA) at The National Center for Biotechnology Information (NCBI) under the accession number SAMN12583022-SAMN12583029. Qiime2 V.2018.11 (Rideout et al., 2018) pipeline was used 16S amplicon data analysis. Quality check and chimera removal of 16S amplicon reads were performed by the dada2 command. Taxonomic classification was performed by consensus-blast command against Silva ribosomal databases v132 (Quast et al., 2013).

## 3 Results and discussion

### 3.1 MFC performance and power generation

**Fig. 1:**
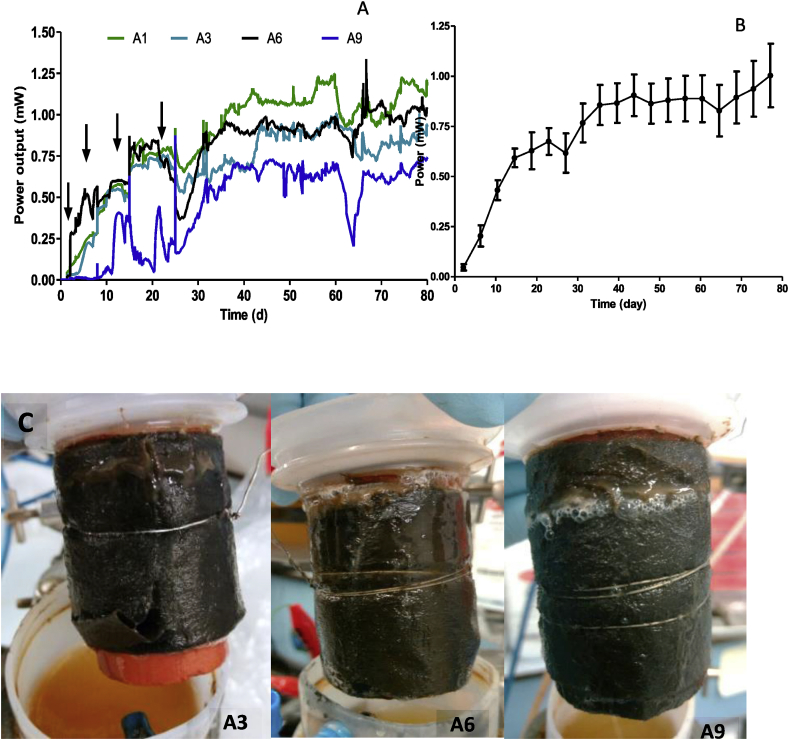
**A**; MFC cascade performance in the first 35 days of operation; arrows show stepwise external load adjustment. **B**; power performance after 80 days of operation where extensive biofilm development was observed. **C**; complete colonisation of the anode surfaces of MFC A3, A6 and A9 by electroactive bacteria community developed without sludge inoculation.

Power was generated after 24 h with the connection of a 700 Ω external load. Further stepwise reduction of the external resistance occurred on day 7 with 400 Ω, day 14 with 200Ω and day 21 with 100 Ω. Changes of the external resistance value resulted in a gradual increase in power generation over time and by day 35, some of the individual MFCs reached more than 1 mW of power (see Fig. 1A), which is similar to the results obtained by identical MFCs inoculated with sludge (Gajda et al., 2015; Winfield et al., 2012). The cascading effect appears to influence power generation as the results showed that the performance of the individual MFCs was relative to the position in the cascade. MFCs in the top half of the cascade produced more power generally than those in the bottom half. This is likely because the MFCs in the top half received more nutrient-rich urine feedstock than those at the bottom, which were receiving the depleted 'digest' from the upstream MFCs. At least 7-day intervals were maintained between external load change to allow sufficient adaptation and development of the bacterial community to the new external load. Fig. 1A&B, show the individual power output by the MFCs in cascade, as well as the average power generation from all nine MFCs, which was an indication of healthy power generation across all individual MFCs. The images in Fig. 1C, show the biofilm developed on the anode of MFC A3, A6 and A9. Stepwise adjustment of the external resistance of urine-fed MFC cascade resulted in the formation of a well-developed bacterial community after 80 days of operation. These results show that the anodic biofilm harboured sufficient amount of electroactive bacteria with the ability to generate an average power of 1 mW, in absolute terms.

Fig. 1B shows long-term power generation by the urine-inoculated MFCs cascade during 80 days of operation time. The results show that the development of the electroactive bacterial community was enough to reach an average of 1.05 mW of power. This is the highest power output recorded by this type of MFC set-up, independent of feedstock. The gradual adjustment of the external resistance of the urine-fed MFCs cascade resulted in the formation of a well-developed *in situ* bacterial community (Katuri et al., 2011). Power generation from this urine-inoculated cascade was greater than the values obtained from MFC cascade of similar configuration which was inoculated with activated sludge (Ieropoulos et al., 2019).

**Fig. 2:**
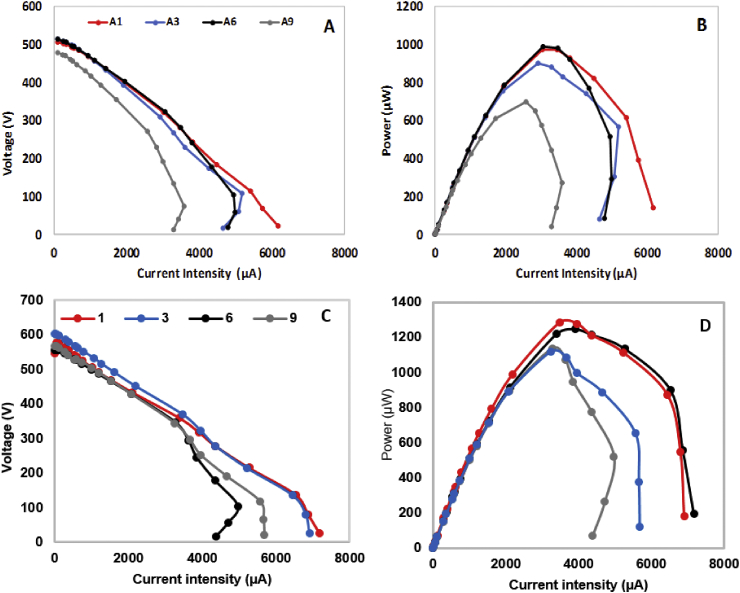
Polarisation (A) and power curves (B) of A1, A3, A6 and A9 systems inoculated with urine and Polarisation (C) and power curves (D) of sludge inoculated cascade conducted after 20 days of operation.

Fig. 2A shows the polarisation curves of the position A1, A3, A6 and A9 of the MFCs cascade inoculated with urine. As can be seen, there are two very different groups. One comprising MFCs A1, A3 and A6, and the second one MFC A9. The first group show a similar slope of the curve in both the activation and ohmic losses region. However, MFC A3 and A6 show higher mass losses unlike MFC A1. In terms of power output, MFC A1, A3 and A6 reached higher values than MFC A9, possibly due to the depletion in substrate as it flows downstream the cascade. The maximum power output follows the same trend for long-term performance and in all cases MFC A9 reached the minimum power output of the cascade (0.697 mW).

Despite the similar performance observed in the case of the MFCs inoculated with sludge and fed with urine, to those inoculated and fed only with urine, in the case of using sludge it was clearly distinguished two groups (Fig. 2C&D). The results show that the MFCs at the start of the cascade reach higher values of power output than those placed in the mid-point onwards, being maximum in the case of the MFC 3 (1.28 mW). The trend of these results is in line with those reported previously in the literature for a cascade of MFCs fluidically connected and fed with artificial wastewater (Winfield et al., 2012). Moreover, in both cases, considerable overshoot in power was observed, which increases with the unit position in the cascade. This phenomenon is caused by the limiting electron transfer at the anode when the potential in the anode rapidly increases while the resistance to current flow decreases. This results in a reduction of the microbial response to the new external resistance applied (Watson and Logan, 2011). It has also been reported that a reduction in the anolyte conductivity might negatively affect the performance of MFCs, causing higher overshoot in power (Winfield et al., 2011). In the case of a cascade of MFCs fluidically connected like the ones used in this work, the urine is treated while flowing from one MFC to the next which reduces the amount of nutrients and therefore the conductivity of the substrate (Winfield et al., 2011).

### 3.2 Microbial ecology of the inoculum and developed anodic bacterial community

#### 3.2.1 Bacterial community composition of the urine feed

**Fig. 3:**
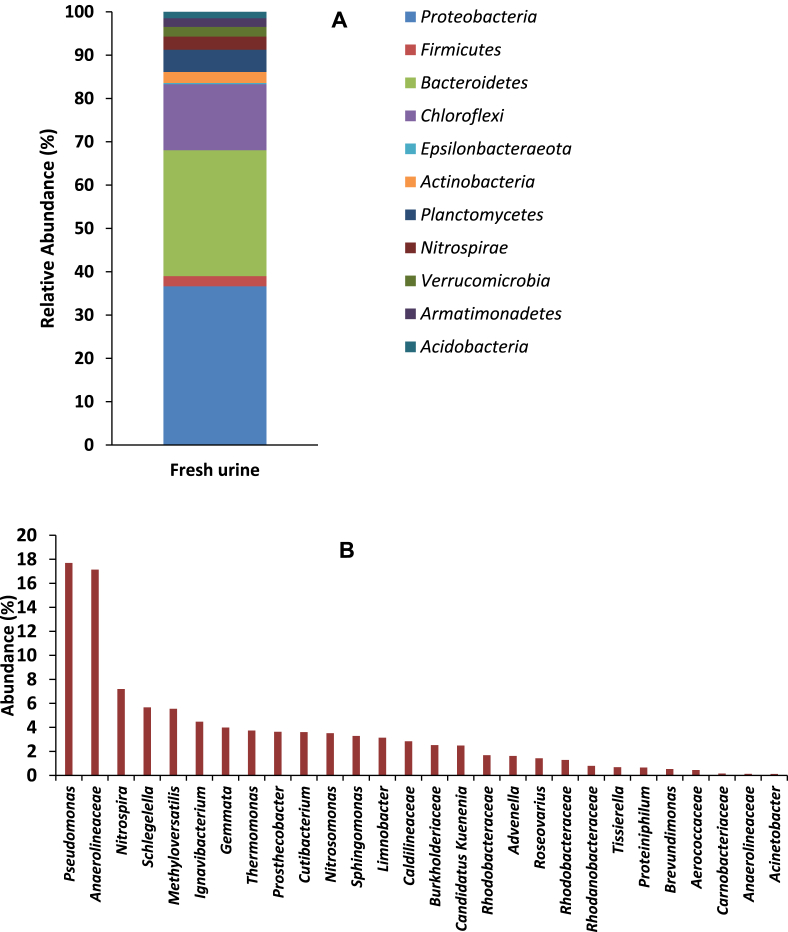
(A) Phyla distribution and (B) genera abundance of the microbial community detected in fresh urine with which the MFC cascade was fed.

To gain an insight into the microbial composition of the urine feed, its microbial community was analysed using 16S rRNA gene sequence approach. The results show a significantly diverse bacterial community, both at phylum and genus level. At phylum level for instance, eleven distinct communities were detected, which were dominated by *Proteobacteria* (37%) and *Bacteroidetes* (29%), followed by *Chloroflexi* (15%) *Planctomycetes* (5%), with the other smaller communities making up less than 14% (Fig. 3A).

*Proteobacteria* which are the dominant bacterial group in the urine feedstock, are also the largest and most phenotypically diverse division of bacteria in nature. They are made up of five classes, which are all known to be gram negative and are commonly found in aerobic conditions (Williams et al., 2019). *Bacteroidetes,* the second largest group, is also a vast bacteria phylum, with significant diversity at different levels of resolution. They are highly adapted to rapidly changing environments such as the human gut. They are the largest bacteria community found in the human gut where they provide humans with energy harvested from fermentation of indigestible polysaccharides (Gibiino et al., 2018; Johnson et al., 2016; Koliada et al., 2017).

*Choloflexi*, the third largest group within the urine feed, are a group of green, non-sulphur, filamentous bacteria which are abundant in activated sludge of wastewater treatment plants (Hugenholtz et al., 2019). They are mainly active under aerobic conditions and are able to utilise sugars, butyrate and some short-chained fatty acids (Kragelund et al., 2006).

Analysis of the bacterial community of the urine feed at the genus level revealed a phylogenetic diversity of at least 28 genera. Members of *Pseudomonas* and *Anaerolinaeae* were the dominant groups, accounting for 18% and 17% of the community, respectively (Fig. 3B). *Pseudomonas* species including *aeruginosa* are highly adaptive in nature, with the ability to colonise different environments. Some species of *Pseudomonas* are however human opportunistic pathogens, causing urinary tract infections (Tashiro et al., 2013; Vital-Lopez et al., 2015). The presence of *Pseudomonas* species in the analysed urine could be a result of colonisation or infection of the urethra of the donor(s) (Mittal et al., 2009; Novoa et al., 2017). Incidentally, earlier reports indicate that members of the genus *Pseudomonas* can produce compounds such as phenazine pyocyanin, which functions as an electron shuttle to an electron acceptor. As such, their presence could aid the power generation processes within the MFC cascade (Rabaey et al., 2004).

Members of the *Anaerolinaeae* spp, which are also dominant in the urine feed have been identified as non-motile, non-sporulating, gram-negative fermentative bacteria. They possess cellulolytic ability and cellular adhesiveness, and are commonly found in anaerobic environments (Xia et al., 2016). Their introduction into the urine-fed MFC could potentially contribute to hydrolysis of urine as well as cellular adhesion within the biofilm matrix.

Other prominent bacterial communities within the urine feed include members of the genera *Nitrospira* and *Methyloversatilis*, which made up 7 and 6% of the community, respectively. The genus *Nitrospira* represent the most diverse group of nitrite-oxidizing bacteria. They represent a key component of the nitrogen-cycling microbial communities, including ammonia oxidation. The unique genomic features of *Nitrospira* perhaps accounts for its competitive success in most nitrifying environments and its adaptation to hypoxic ecosystems (Daims et al., 2015; Lücker et al., 2010). Their presence could be linked to the ammonia component of urine and their involvement in ammonia oxidation.

Members of *Methyloversatilis* on the other hand, show a wide range of metabolic abilities and are able to utilise several organic acids, alcohols as well as methanol and methylamine. They thrive in pH range 6.6–8 but are not able to survive at pH below 4 or above 8.5 (Kalyuzhnaya et al., 2019; Smalley et al., 2019). Their narrow survival pH range would have implications for its ability to thrive in urine fed MFCs operated at pH > 9. Changes in pH and conductivity across MFCs within the cascade are shown in Fig. S2.

#### 3.2.2 Microbial composition of the established anodic community within urine inoculated MFCs after 80 days

**Fig. 4:**
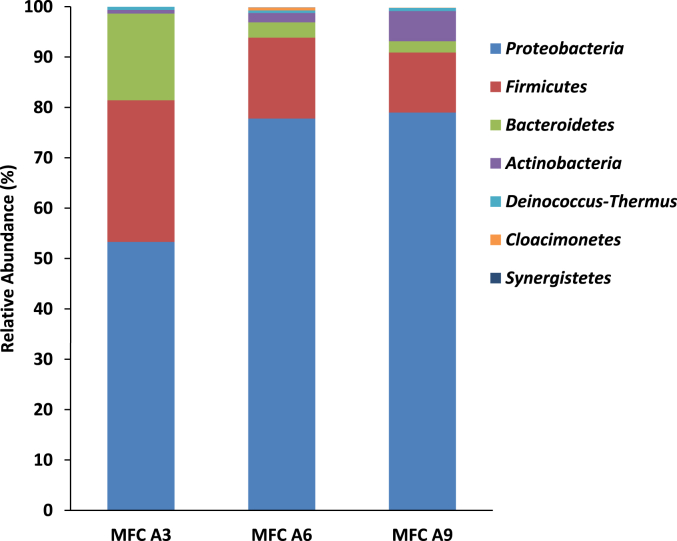
Phyla distribution of the microbial community detected in the anodic biofilm pf MFC A3, A6 and A9 after 80 days of operation.

After the successful development of anodic bacterial community and the attainment of steady power generation, samples were collected for microbial analysis to evaluate the microbial ecology of the MFC cascades. Analysis of the developed bacterial community on the anode after 80 days revealed a diverse community, with at least seven different phyla detected. Fig. 6 presents community distribution of the anodic bacteria at the phylum level and highlights the dominance of bacteria belonging to the phylum *Proteobacteria* within MFC A3 (53%), MFC A6 (78%) and MFC A9 (79%). Bacteria related to the phylum *Firmicutes* represent the second largest community of the developed anode, accounting for 28%, 16% and 12% bacterial community of MFC A3, A6, and A9 respectively (Fig. 4).

*Bacteroides*, which formed 17% of the bacterial community in MFC A3, accounted for only 3% and 2% within MFC A6 and A9 respectively. On the other hand, the phylum *Actinobacteria*, which formed below 1% in the bacterial community within MFC A3, accounted for 2% and 6% for MFC A6 and A9 respectively (Fig. 4). The results showed that MFC A6 and A9 harboured more diverse communities than MFC A3, and indicate that bacterial community distribution was dependent on the position of the MFC within the cascade, which is perhaps the result of metabolic rate and nutrient composition within each MFC.

Previous research has reported on the dominance of bacteria belonging to the phyla *Proteobacteria* and *Firmicutes* within anodic biofilm of various MFC systems (Chae et al., 2009; Rabaey et al., 2004). This is not surprising as most of the currently identified electroactive bacteria such as *Geobacter* and *Shewanella* sp. belong to the phylum *Proteobacteria* (Paitier et al., 2017; Suzuki et al., 2018; Zhang et al., 2011). One of the very few studies of the anodic microbial community composition of urine fed MFC stacks showed that *Proteobacteria* and *Firmicutes* were the dominant phyla within systems [48]. This report also highlighted the impact of nutrient type as well as dissolved oxygen concentration on the distribution of the microbial community (Cid et al., 2018). The detection of aerobic, anaerobic and facultative bacteria in the anodic biofilm of these MFCs is an indication of stratification that exists within the anode chamber, where the top of the chamber was aerated while the bottom was mostly anoxic (Walter et al., 2016a).

**Fig. 5:**
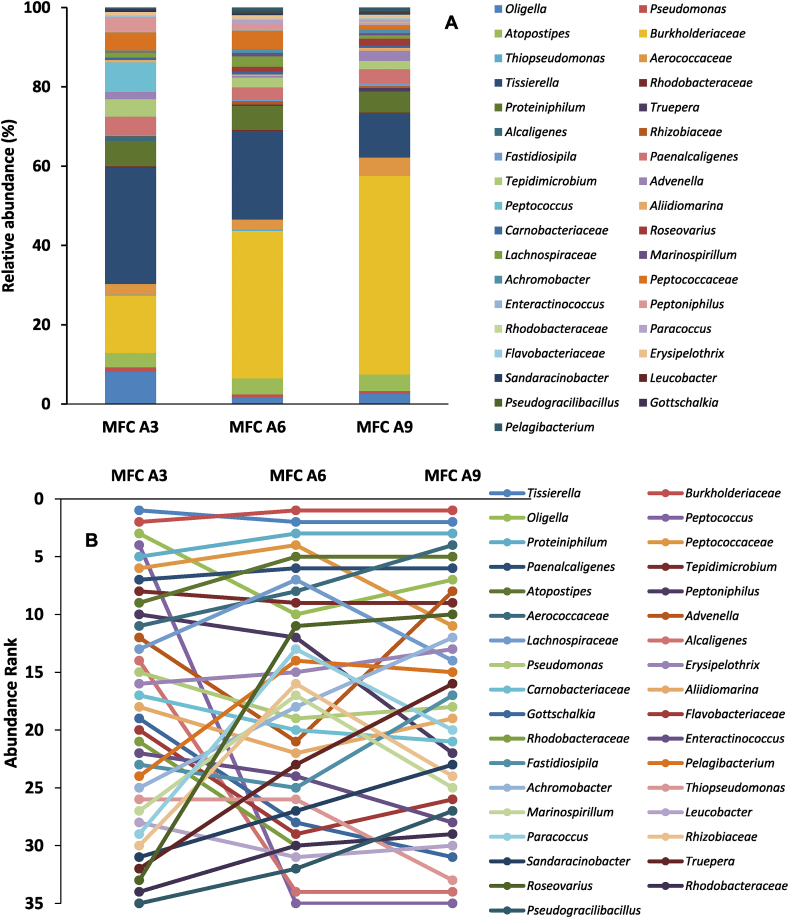
Relative abundance (A) and Genus ranking (B) of different bacteria strains on the developed biofilm of individual **urine-inoculated** MFC A3, A6 and A9 at the genus level.

Analysis of the community to the genus level showed a highly diverse community with at least 35 genera identified in MFC A3, A6 and A9 (Fig. 5A). The genera *Tissierella* and *Burkholderiaceae* dominated the communities in the three MFCs analysed. *Tissierella* was the dominant community making up of almost 30% of the bacterial community in MFC A3, 22% in MFC A6 and 11% in MFC A9. Moreover, members of *Burkholderiaceae* constituted half (50%) of the bacterial community in MFC A9, which was at the bottom of the cascade compared to 37% and 15% in MFC A6 and MFC A3 respectively (Fig. 5A). Essentially, while the proportion of the genus *Tissierella* declines down the cascade, the proportion of *Burkholderiaceae* increased down the cascade. This might be due to the gradual urine degradation and differences in the nutrient availability within individual MFC. This finding is in agreement with previous reports that nutrient type and concentration were strong determinants of microbial community structure in an MFC (Chae et al., 2009; Park et al., 2017b).

**Fig. 6:**
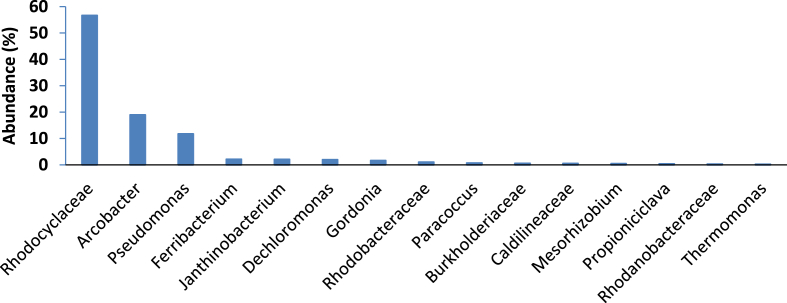
Relative abundance of various bacterial community detected in anaerobically digested sludge used for MFC inoculation. Communities >0.2% shown.

Meanwhile, members of the genus *Pseudomonas,* which dominated the bacterial community within the urine feed (18%) at the genus level (Fig. 3B), was reduced to less than 1% in the MFC cascade after 80 days of operation (Fig. 6). Furthermore, members of *Anaerolinaceae*, which constituted 17% of the bacterial community in the urine feed, was below detection levels within the established EAB on the anodes. This reduction in numbers might be because of their lack of involvement in the hydrolytic and bio-electrochemical processes within the MFC cascade. Meanwhile, bacteria related to the genus *Tissierella,* which accounted for less than 1% of the microbial community of the urine feed, was one of the most dominant genera (up to 30%) within the EAB community of the developed anode after 80 days. This again is an indication of the selection, enrichment and adaptation of microbial communities within MFCs, as a result of inherent nutrient composition and *in situ* reactions (Rabaey et al., 2004).

Although there were changes in the proportion of some of the microbial communities along the cascade (Fig. 5A), some of the genera within the three MFCs remained relatively unchanged. For instance, the genera *Atopostipes* of the phylum *Firmicutes* maintained a proportional composition of 4% in each of MFC A3, A6 and A9 (Fig. 5). *Atopostipes* are gram positive, facultative anaerobic bacteria, with an optimum growth temperature of 28–30 °C. They produce lactate and acetate from the glucose metabolism, but have no known urease activity (Cotta et al., 2004). They are perhaps not affected by the changes in the substrate composition along the cascade, hence their stability.

Other genera with consistent community representation within all three MFCs were *Paenalcaligenes* (~4%) *Aerococcaceae* (~3%) *Proteiniphilum* (~6%). Some of these consistent communities might be important for process and functional stability of the individual MFC reactors (Fernandez et al., 2000).

These results of the microbial community showed significant differences as well as increases in diversity between the bacterial community of the urine feedstock and those obtained in the well-developed anodes (after 80 days). The microbial community of the developed anode possesses greater diversity (35 genera) (Fig. 5A) than that of the fresh urine (28 genera) (Fig. 3B). The results suggest that a process of selection and adaptation brought about the development of an efficient community of bacteria working together to achieve urine metabolism leading to stable power generation. Several factors might be responsible for the selection of the various microbial communities in the urine-inoculated MFCs within the cascade. Some of these include the type and composition of the substrates, pH, conductivity as well as the stratification within the anode chamber (Johnson et al., 2016; Rabaey et al., 2004; Rodyou et al., 2017; Walter et al., 2016a).

Some of the bacterial strains that were undetected in the fresh urine were found in the developed biofilm community and vice versa. An example was the *Methyloversatilis*, which was the fourth largest group in the urine feedstock (6%), but went below detection limits in the developed anodic biofilm after 80 days. A reason for the decrease of this genus could be the drastic increase in pH above 8.5 in all three MFCs, a pH above which *Methyloversatilis* could not survive (Kalyuzhnaya et al., 2019; Smalley et al., 2019).

The abundance of bacteria related to the genus *Tissierella* in all MFC analysed shows their importance to urine metabolism and overall power generation processes. Earlier reports have shown that members of *Tissierella* utilise creatinine, which is an important component of urine as their sole carbon source. In most *Tissierella*, creatinine metabolism occurs via N-methylhydantoin, N-carbamoyl-sarcosine, and sarcosine to acetate, ammonia, and CO_2_. Acetate generated by members of *Tissierella* could be the main carbon source for other bacterial strains within the community which readily metabolise acetate such as *Erysipelothrix* (Menon and Voordouw, 2018) and *Peptococcus* (Ato et al., 2014).

The family *Burkholderiaceae* on the other hand contain many strains, which are known electroactive bacteria and could be responsible for power generation in the MFC cascades under investigation. Examples of known electroactive bacteria within *Burkholderiaceae* include *Rhodoferax sp* and *Cupriavidus sp*., both of which carry out extracellular electron transfer through both direct and mediated means to electrode surfaces (Sydow et al., 2014). Meanwhile research has shown that members of *Burkholderiaceae* dominate acetate enriched MFCs (Borole et al., 2009), and as such there are strong indications that the acetate generated by members of *Tissierella* was utilised by members of *Burkholderiaceae*. This could account for the inverse relationship between the prevalence of these 2 bacterial strains along the cascade. Essentially, the proportion of *Burkholderiaceae* increased along the cascade (Fig. 5A) as more acetate was made available by members of *Tissierella* which in turn decreased in numbers as the concentration of creatinine on which they depend declined along the cascade.

The diversity index within individual MFCs of the urine-inoculated cascade was shown in Fig S5. It shows that MFC A3 contained significantly more diverse bacterial community than MFCs A6 & A9.

The overshoot phenomenon discussed earlier was minimal in MFC A3 and increased along the cascade. Apart from the variations observed in the proportions of the bacterial community of different MFCs within the cascade (Fig. 6, Fig. 8), a notable difference was recorded between the community structure of MFC A3 and MFC A6 and A9. The community analysis showed the absence of 2 important genera in MFC A6 and A9 which were present in MFC A3. These include *Peptococcus* and *Alcaligenes* which constitute 8% and 1.3% of the MFC A3 respectively. Members of *Peptococcus* are avid acetate metabolisers (Ato et al., 2014) while *Alcaligenes* strains possess the unique ability to metabolise ammonium and possess nanowires for potential transfer of electrons externally to electrode surfaces (Wang et al., 2015). The lack of activity from these 2 absent genera, especially the additional electron transfer capability of *Alcaligenes*, coupled with slightly lower conductivity (Fig. S2) and lower nutrient availability in MFC A6 and A9, perhaps resulted in the considerable overshoot recorded in those MFCs.

### 3.3 Microbial analysis of sludge inoculum and sludge-inoculated cascade

#### 3.3.1 Bacterial community composition of the sludge inoculum

Fig. 6 showed the relative abundance of the bacteria community found in the sludge inoculum, with the detection of at least 27 different bacterial strains. The results show the dominance of bacteria related to *Rhodocyclaceae* which, make up 57% of the community whilst *Arcobacter* and *Pseudomonas* make up 19% and 12%, respectively. The remaining 24 smaller communities make up only 12% of the bacterial community of the sludge inoculum (Fig. 6).

Members of *Rhodocyclaceae,* which dominated the sludge inoculum are widespread in nature. They have been detected in various environments including soil, sewage treatment plants and polluted water. *Rhodocyclaceae* is made up of 45 different species, thus exhibiting a diverse mode of survival and utilising a wide range of carbon sources. Members of *Rhodocyclaceae* are sulphur-oxidising chemoautotrophs, methylotrophs, and anaerobes that perform propionic acid fermentation (Oren, 2014). It is thus not surprising that they dominate the communities in the sludge inoculum obtained from wastewater treatment plant. *Arcobacter* on the other hand are gram negative, with 6 species which are able to grow under aerobic and anaerobic conditions (Ho et al., 2006; Pérez-Cataluña et al., 2018), conditions obtainable within the anodic chamber of the MFCs.

#### 3.3.2 Bacterial community composition of the anodic community of the sludge inoculated MFCs after 80days of operation

**Fig. 7:**
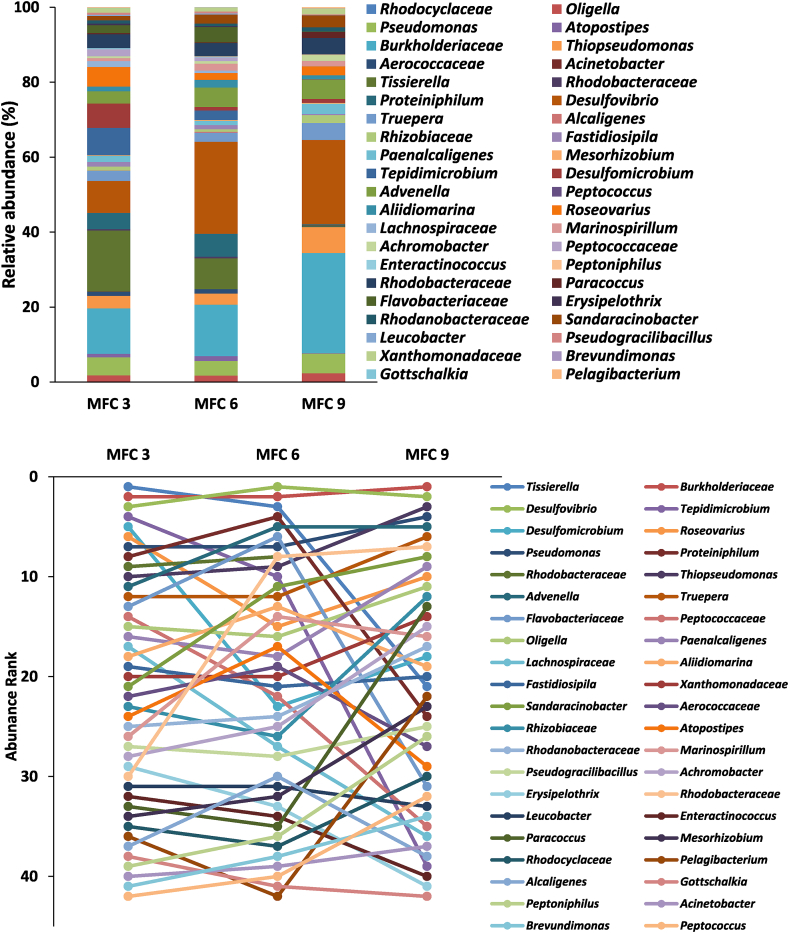
Relative abundance (A) and Abundance ranking and changes in the dominance (B) of various bacterial community (at genus level) detected in the anode of sludge-inoculated MFC cascade after 80 days of operation.

Analysis of the bacterial community of the developed anodic biofilm showed a significant diversity within the bacterial community with the detection of at least 42 genera. MFC 3 was dominated by members of genera *Tissierella* (16%), *Burkholderiaceae* (12%) and *Desulfovibrio* (8%). MFC 6 was dominated by *Desulfovibrio* (25%) *Burkholderiaceae* (14%) and *Tissierella* (8%) while the proportions of dominant *Burkholderiaceae, Desulfovibrio* and *Thiopseudomonas* in MFC 9 were 27%, 23% and 7% respectively (Fig. 7). At a glance, the results showed that the developed anodic biofilm of the sludge inoculated cascade was almost twice as diverse as the sludge inoculum. Members of *Rhodocyclaceae* which dominated the sludge inoculum (57%) was reduced to less than 1% in all MFCs after 80 days of operation. *Arcobacter,* which constituted 19% of the bacteria in the inoculum, was below detection within the anodic biofilm. In general, there are 14 similar genera between the sludge inoculum and the developed biofilm communities, whilst 13 different genera detected in the inoculum were below detection in the biofilm. Meanwhile, 28 of the bacteria strains detected in the biofilms were not found in the sludge inoculum.

**Fig. 8:**
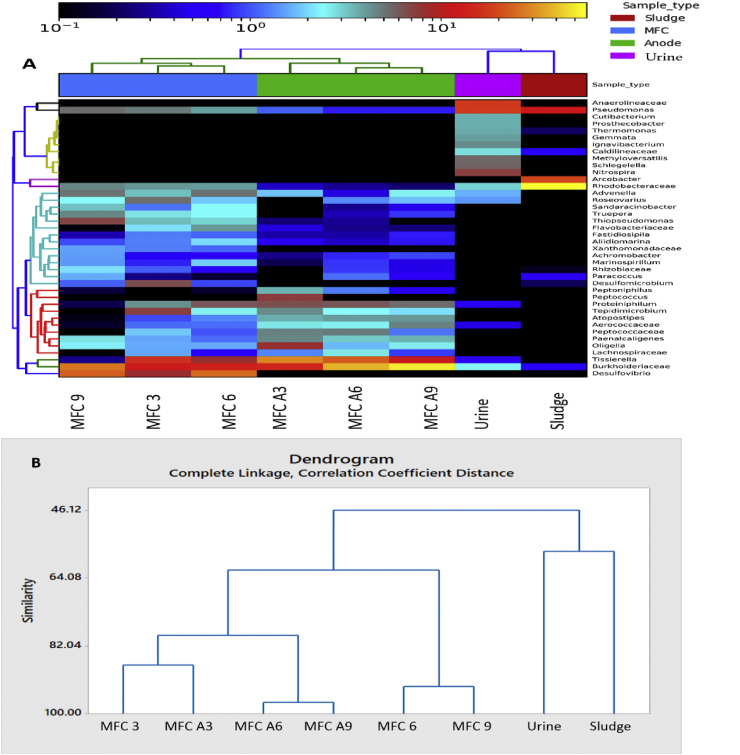
Heat map (A) and Dendogram (B) construction showing the similarities among the different bacterial communities of the inoculums, sludge and urine inoculated MFCs.

Comparing the bacterial community of the urine feed and developed anodic biofilm showed that 14 of the communities in the urine feed were similar to those of the anodic biofilm of the sludge inoculated cascade, and as such the urine feed perhaps contributed significantly to the enrichment of the anodic community in addition to the sludge inoculum.

This observation is consistent with previous research, which showed significant differences between the microbial community of the inoculum and the developed anodes of MFCs (Suzuki et al., 2018).

Certain trends observed in the community structure of the urine-inoculated cascade was also evident in the sludge inoculated MFCs. For instance, increases in the abundance of *Burkholderiaceae* along the cascade with the corresponding decline in the proportion of *Tissierella* was observed (Fig. 7A&B). The bacterial community changed substantially along the cascade, depending on the position of the MFCs (Fig. 7B). This is perhaps affected by the differences in the microenvironment within the anodic chamber of individual MFCs, caused by factors such as changes in nutrient composition.

Although greater diversity was recorded in the bacterial community of the sludge inoculated cascade (Fig. 7A), there was a significant similarity in the strains of bacteria detected in both urine and sludge inoculated MFCs. Essentially, 34 of the 35 different strains found in the urine-inoculated MFCs were also detected in the sludge inoculated MFCs. There are however some important differences between the two set ups. One is the dominance of bacteria belonging to the genus *Desulfovibrio* (up to 25%) in the sludge inoculated MFCs which was completely absent in the urine-inoculated MFCs.

The genus *Desulfovibrio* consist of bacterial strains that can utilise many organic acids, alcohols or hydrogen for sulphate reduction. They have been reported as important electroactive bacteria with the ability to carry our direct extracellular electron transfer (Lu et al., 2019; Zhang et al., 2019). One species, *D. desulfuricans* utilises lactate as its carbon source and sulphate as electron acceptor. Sulphide generated from sulphate reduction is electrochemically active at the anode where it is oxidised to sulphate with concomitant release of electrons, giving rise to greater power density in MFCs due to cells not requiring to synthesise endogenous mediators (Habermann and Pommer, 1991; Ieropoulos et al., 2005). The contribution of this bacterial strain perhaps facilitated efficient electron transfer at the earlier stages of the sludge-inoculated MFCs operation, leading significantly to higher power generation (Fig. S1).

Fig. 8 (A&B) show the differences and similarities among all the bacterial community analysed. At a glance, the results revealed a significant difference between the two inocula, whilst the developed anodic community of the 2 cascades showed substantial similarities. For instance, although MFC 3 and MFC A3 were inoculated differently, the bacterial community of the developed anode was more than 85% similar.

The prevalence of different strains of bacteria within the anodic biofilms of urine and sludge inoculated cascades demonstrates the importance of microbial interactions and cooperation for substrate degradation and concomitant electricity generation within individual MFC (Tashiro et al., 2013). Even those organisms, which are perhaps not directly involved with electron transfer such as fermenters, must have aided the activities of the electroactive community by making useable nutrients available. The substrates utilised in this study might have influenced the type of electroactive bacteria present on the anode. Substrate types have been shown previously to be as a strong determinant of the composition of the microbial community as well as the MFC performance including power density in MFCs (Sydow et al., 2014). For instance, in an acetate enriched MFC, *Geobacter* was the most detected genera while *Bacillus* *sp* dominated MFCs enriched with propionate. Furthermore, the anodic community glucose enriched MFC have also been shown to be dominated by *Pseudomonas* *sp* (Rabaey et al., 2004; Sydow et al., 2014). The low proportion of *Pseudomonas* within the anode community of MFCs in the current study, is perhaps an indication of low glucose concentration in the urine feed. Although members of the well-known electroactive bacteria such as *Shewanella* and *Geobacter* *sp*. were not detected in the current study, there are indications that other less known EAB communities belonging to the phylum *Firmicutes* and family *Burkholderiaceae* e.g. *Rhodoferax sp* and *Cupriavidus sp*. might be responsible for the recorded power generation (Sydow et al., 2014; Zhao et al., 2018). Generally, the results show that substrate type (urine), substrate composition and external load application, as well as other inherent factors, could have influenced the selection and adaptation of the anodic microbial community composition and structure.

## 4 Conclusions

The current study highlights the possibility of starting a urine fed MFC treatment system in remote locations without the difficulty of accessing a sludge inoculum. The use of neat human urine as both the inoculum and feedstock brought about the generation of similar power levels in comparison to those inoculated with anaerobically digested sludge. The 16S rRNA gene analysis showed the dominance of *Pseudomonas* and *Anaerolinaceae* like bacteria in the urine feed, while members belonging to the genera *Burkholderiaceae* and *Tissierella* dominated the bacterial community of the developed anode after 80 days of operation. The results demonstrate a selection and adaptation process occurring within the cascade due to the substrate type, external load, position in cascade and other inherent bio-electrochemical processes within individual microbial fuel cells.

## Author contributions

Conceptualization: II, OO. Data curation: OO, II. Formal analysis: OO, HK, KC.Funding acquisition: II, JG. Investigation: OO, MJS, HK. Methodology: OO, MJS, HK. Project administration: II, JG, KC. Supervision: II, JG, KC. Validation: OO, II, JG, KC. Visualization: OO, II, HK. Writing – original draft: OO, MJS, HK. Writing – review & editing: OO, II, MJS, HK. The authors declare no conflict of interest for this manuscript.
