# Supplementary material for: Development of efficient electroactive biofilm in urine-fed microbial fuel cell cascades for bioelectricity generation
Source: J Environ Manage. 2020 Mar 15;258:109992. doi: 10.1016/j.jenvman.2019.109992 (PMC7001104; doi:10.1016/j.jenvman.2019.109992)
Supplement: Multimedia component 2 [file mmc2.docx]

**Supplementary data**


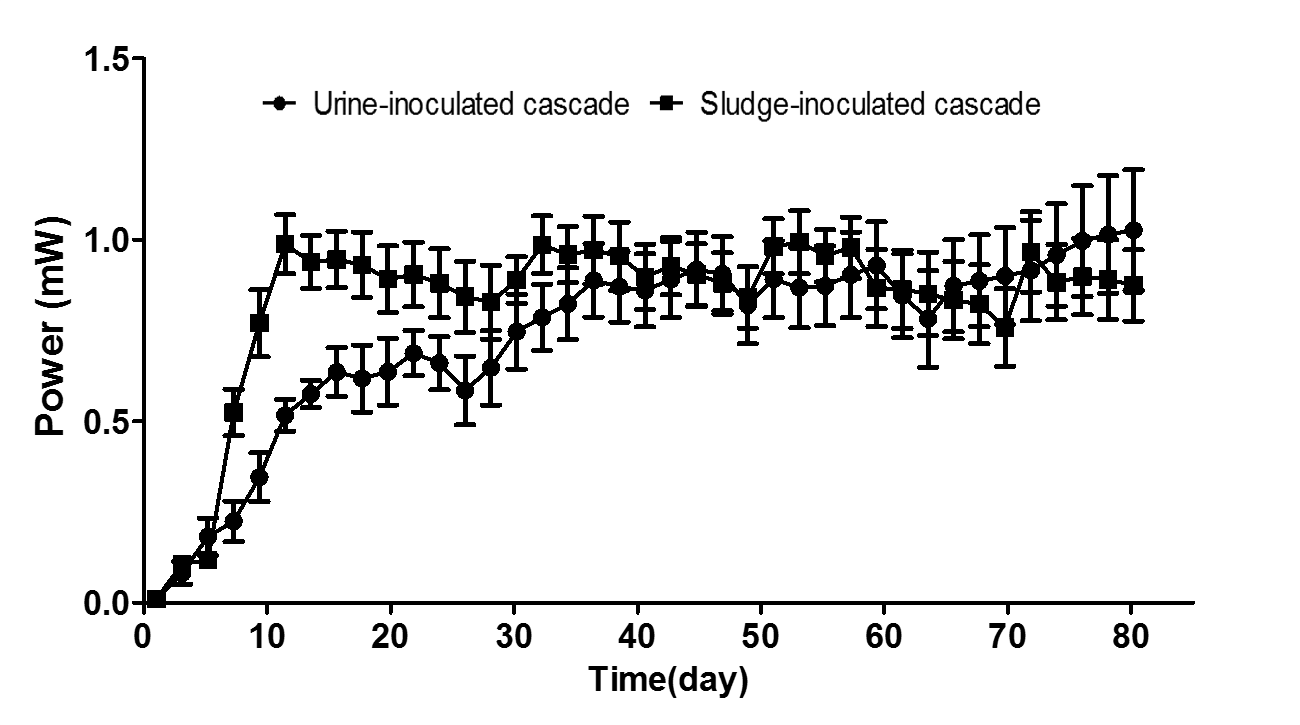


Fig S1. Average long term power generation from the urine and sludge inoculated cascades.

Fig. S2. pH and conductivity within urine feed and urine-inoculated MFCs across the cascade.

Figure S3. Relative abundance of various bacterial community detected in the sludge inoculum at the phylum level.

Figure S4. Relative abundance (at phylum level) of various bacterial community detected in the developed anode of sludge-inoculated MFC cascade after 80 days of operation.

Figure S5. Dominance and diversity index of bacterial community of individual **urine-inoculated** MFCs within the cascade

Figure S6. Dominance and diversity index of bacterial community of individual **sludge-inoculated** MFCs within the cascade
